# Recurrent epistaxis from inflamed granulated tissue and an associated pseudoaneurysm of the internal carotid artery: case report

**DOI:** 10.1186/s12883-021-02254-0

**Published:** 2021-06-03

**Authors:** Ja Yoon Kim, Yong Bae Kim, Joonho Chung

**Affiliations:** 1 Yonsei University College of Medicine, Seoul, Republic of Korea; 2 Department of Neurosurgery, Severance Hospital, Yonsei University College of Medicine, 50-1, Yonsei-ro, Seodaemun-gu, Seoul, 03722 Republic of Korea

**Keywords:** Epistaxis, Granulation, ICA pseudoaneurysm, Brain abscess, Case report

## Abstract

**Background:**

Chronic inflamed tissue in nasal cavity is a rare complication of transsphenoidal approach (TSA). Inflamed tissue is rich in blood vessels, which can lead to frequent nosebleeds. In addition, chronic inflammation can cause pseudoaneurysm, whose rupture results in massive epistaxis. There have been few reported cases of pseudoaneurysm of ICA occurring more than 10 years after TSA surgery.

**Case presentation:**

We report a case of a patient who had recurrent epistaxis for over a decade after TSA surgery, and analyzed the causes of the nosebleeds. The aspect of occurrence of the nosebleeds and the result of biopsy and imaging tests suggest that the nosebleeds were due to chronic inflamed tissue and an associated pseudoaneurysm. The rupture of pseudoaneurysm recurred after treatment with stent placement, and brain abscess was developed. After removing the inflamed tissue by endoscopic resection, the patient no longer had recurrence of ruptured pseudoaneurysm or nosebleeds.

**Conclusions:**

In patients with recurrent nosebleeds, the possibility of intranasal inflammation and subsequent pseudoaneurysm should be considered. Therefore, people who consistently have epistaxis after TSA, even if the bleeding is not in large amount, should be actively screened and treated for nasal chronic inflammation.

## Background

Massive epistaxis from a ruptured pseudoaneurysm of the internal carotid artery (ICA) is a rare but serious complication of the transsphenoidal approach (TSA). This complication usually occurs within a few weeks after surgery, and there have been no reported cases of delayed rupture of pseudoaneurysm occurring more than 10 years after surgery. Pseudoaneurysm can be treated by endovascular interventions, such as graft stent insertion and coil embolization, and it rarely recurs after treatment. Here, we describe a case of recurrent epistaxis that occurred more than 10 years after TSA surgery, and discuss the effects of chronic inflammation in the formation of a pseudoaneurysm and the development of brain abscesses.

## Case presentation

A 56-year-old Asian man presented with recurrent right-sided epistaxis. The patient had a history of hypertension and hypothyroidism as well as a surgical history of subtotal resection of a pituitary adenoma by TSA on January 2006. Since then, the tumor size has remained stable on follow-up magnetic resonance imaging (MRI).

**Fig. 1:**
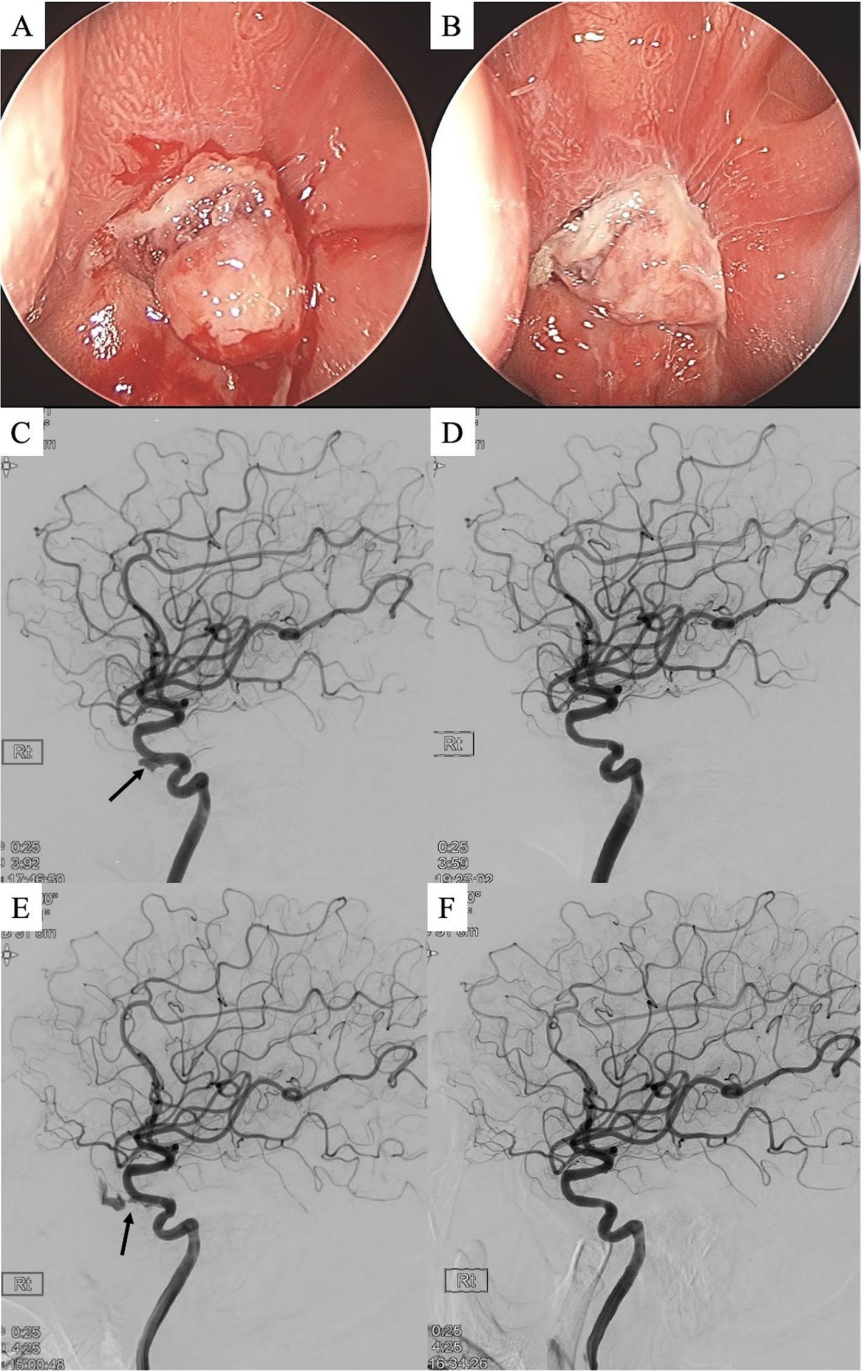
Nasal endoscopy from the patient’s first visit for epistaxis. Nasal polypoid mass **a** before and **b** after cauterization. The first pseudoaneurysm rupture **c** before and **d** after graft stenting on the right internal carotid artery angiography. Repeat rupture of the pseudoaneurysm **e** before and **f** after additional graft stenting. *Black arrow indicates pseudoaneurysm of the internal carotid artery.*

In 2018, the patient visited the hospital complaining of recurrent epistaxis. The patient had intermittently suffered nosebleeds and blood-tinged sputum (which is thought to be posterior nasal drip) after undergoing TSA surgery in 2006, and the symptoms worsened in the latest 1 month; therefore, he decided to visit the hospital. A polypoid mass with an oozing-pattern bleeding in the right sphenoid area was found on navigation-assisted endoscopy, and a biopsy was taken. After endoscopic cauterization, the bleeding seemed to stop (Fig. 1A and B). Histology revealed that the tissue obtained from the biopsy was inflamed granulation tissue. Ten months later, intermittent nasal bleeding resumed. Nine months after, the patient was admitted to the emergency department with about 2L of blood loss due to epistaxis. Emergent endoscopy revealed a bleeding focus on the anterior wall of the right sphenoid, and computed tomography (CT) angiography demonstrated a pseudoaneurysm in the right ICA that recovered from rupture in the area of the previous TSA. A graft stent was inserted into the right ICA pseudoaneurysm (Fig. 1C and D). Four months later, a large nosebleed recurred, and a graft stent was re-inserted for the recurrent pseudoaneurysm rupture (Fig. 1E and F). A CT angiography taken 3 months later demonstrated no evidence of recurrent flow in the treated pseudoaneurysm in the right distal ICA.

**Fig. 2:**
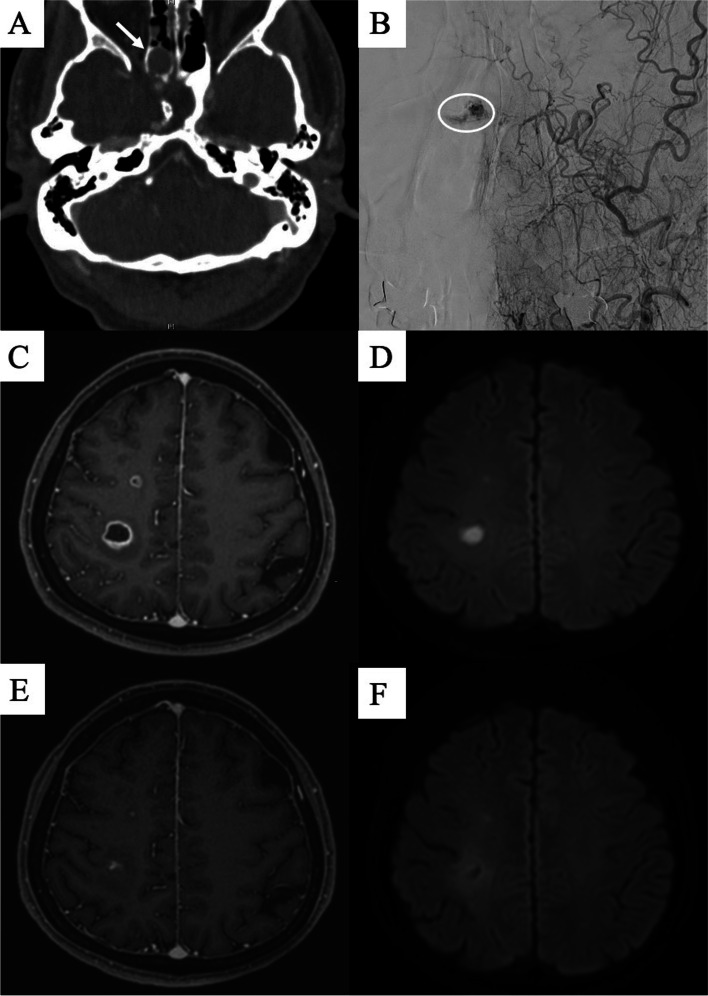
Evidence of chronic inflammation in the right nasal cavity. **a** Granulation ball with slight enhancement on brain CT, *indicated by white arrow*. **b** Left external carotid artery angiography shows a small granulated mass with capillary filling, *indicated by white circle*. Multiple brain abscesses in the right hemisphere show **c** peripheral enhancement on a brain MRI T1-enhanced image and **d** diffusion restriction on a diffusion-weighted image. Brain MRI at the 1-month follow-up visit shows regression of the abscesses on **e** T1-enhanced image and **f** diffusion-weighted image

**Fig. 3:**
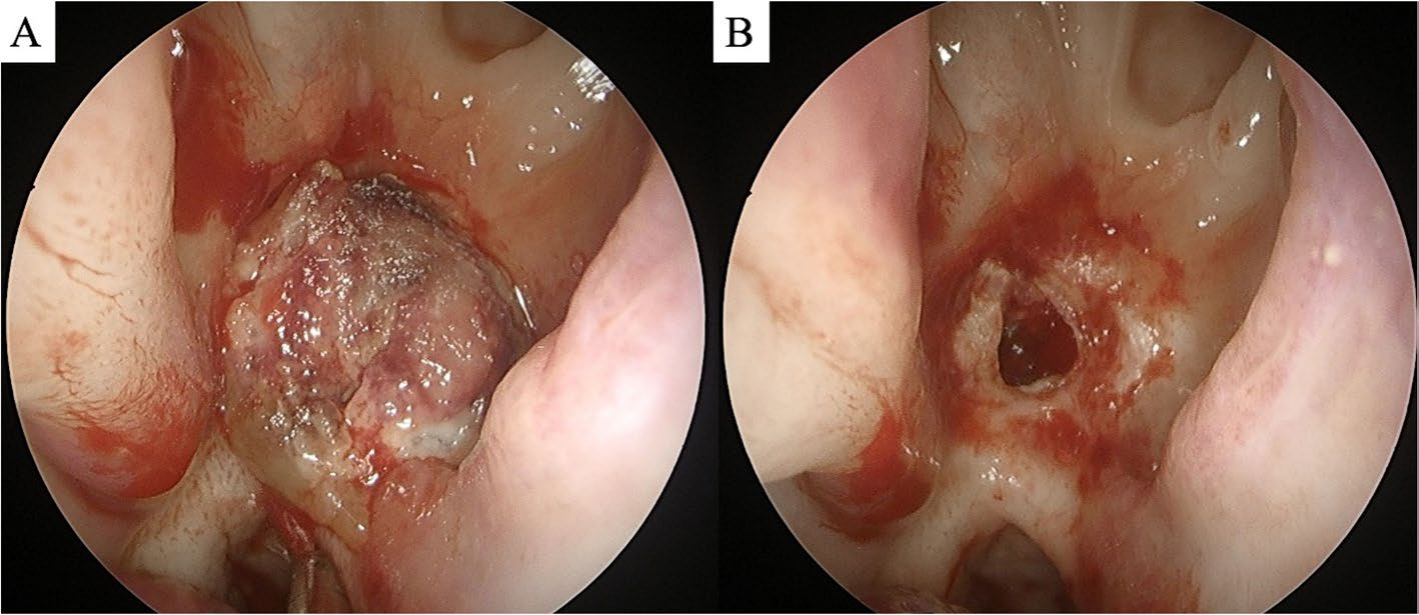
Nasal endoscopy **a** before and **b** after resection of the nasal polypoid mass

Shortly thereafter, the patient returned to the hospital with epistaxis accompanied by fever and sinus tenderness. Peri-stent enhancement on the inferior medial side of the right nasal cavity was noted in a left external carotid artery (ECA) angiogram, and an accordant mass was seen on brain CT (Fig. 2A and B). Furthermore, multiple brain abscesses were observed on MRI (Fig. 2C and D). CSF culture was negative. However, considering that brain abscess may occur over a long time, methicillin-susceptible staphylococcus aureus (MSSA), which was cultured in both blood and sputum (posterior nasal drip) 5 months ago, was estimated as the causative agent. The patient underwent endoscopic resection about the mass, and was treated with the proper antibiotics (Fig. 3). Ciprofloxacin was used in consideration of the patient’s adverse drug reaction, and it was administered orally for 32 days (250 mg 1 T bid). Histology revealed that the resected mass was necrotic mucosal tissue with exudate and blood clots. During the 5-month follow-up period, the operation site was well maintained without further bleeding, and brain MRI showed regressed abscesses (Fig. 2E and F).

## Discussions and conclusions

Epistaxis is an uncommon but known post-operative complication of TSA that generally occurs 1–3 weeks after surgery [1, 2]. Delayed recurrent epistaxis occurring years after TSA for pituitary adenoma is extremely rare. In our case, the patient suffered episodes of epistaxis, with both small and large blood loss, several times long after undergoing TSA surgery. The episodes of epistaxis with minimal blood loss were a result of bleeding from the inflamed granulation mass, whereas the episodes with a large quantity of blood loss were from a ruptured pseudoaneurysm.

Granulation tissue is a highly vascularized tissue that forms as chronic inflammation progresses. Histologically, it appears as pinkish granulated tissue that consists of capillaries. Given that TSA surgery is performed through the nasal cavity, there is a possibility that granulation tissue can form around the wound. The patient was not suffering from any immunosuppressive disease or taking immunosuppressive drugs, and no infectious agents capable of causing inflammation were found in the biopsy. Therefore, it is likely that a granulated tissue was formed as the result of tissue damage during surgery.

The abundant blood vessels can easily burst and cause chronic bleeding. Cauterization of bleeding foci can be performed as conservative treatment; however, if symptoms persist or are severe, the mass can be removed by endoscopic surgery. Considering that the patient in the present case had intermittently shed a small amount of blood from his nose for over a decade after TSA surgery, it is highly likely that the inflammatory mass developed after the surgery. We were able to see the mass on brain CT, and ECA angiography showed that the mass was rich in blood vessels. The pathogenesis of the cerebral abscesses may have been developed by infection from the persistently inflamed granulation tissue, which could have been a possible route for infection to the brain [3].

Pseudoaneurysm occurs when the blood vessel is physically or chemically damaged. Not only for complications of TSA surgery, pseudoaneurysm of femoral artery after catheterization and pseudoaneurysm in cesarean Cesarean section scar pregnancy are known examples [4, 5]. Pseudoaneurysm and its complications, such as ruptures, usually occur immediately after or within a few weeks of vascular injury. Although there have been some previously reported cases of delayed pseudoaneurysm after TSA [6–8], it is very rare for pseudoaneurysm to develop and rupture more than 10 years after blood vessel damage (by TSA surgery), as in our case. Also, pseudoaneurysm is effectively treated with graft stent and coil embolization, and most of the time, they are completely obliterated [9–12]. The recurrence of treated pseudoaneurysm is not common; however, in our case, the previously treated pseudoaneurysm ruptured again 4 months after treatment.

Therefore, in our patient, the pseudoaneurysm may have been caused by either the direct effect of TSA or the effect of adjacent granulation tissue. Considering that pseudoaneurysm occurred after more than 10 years from TSA, the latter has more possibility. Although the original occurrence of pseudoaneurysm might have been a complication of TSA itself, what caused it to rupture and recur seems secondary to chronic local inflammation of the granulation tissue. The ICA pseudoaneurysm in the right cavernous sinus was close enough to be affected by the nearby nasal granulation tissue, and the repeated episodes of inflammation could have led to erosion of the adjacent vessel walls, a theory which is consistent with the patient’s history that the first rupture of the pseudoaneurysm was observed 19 months after active bleeding in the granulation tissue. The fact that splenic artery pseudoaneurysm is a well-known complication of acute/chronic pancreatitis also supports this idea [13, 14].

Another complication due to chronically inflamed tissue in nasal cavity is brain abscess. Therefore, when a patient complains about fever and sinus tenderness, physicians should perform a more detailed examination, not only for the nose but also for a brain infection. In our case, brain abscess biopsy was not performed, and the result of CSF culture was negative. However, in the patient’s blood and sputum, MSSA was cultured. Given that staphylococcus aureus is a representative flora of nasal mucosa [15], and the brain abscess cured with antibiotics for MSSA, it could be assumed that the inflammation in the nose caused brain infection. Also we thought that the sputum mentioned above had originated from posterior nasal drip. Although we should consider that brain abscess occurred as a complication of graft-stent insertion [16], it cannot be completely ruled out that the abscess was caused by chronic inflammation in the nose.

Our case highlights the need to assess chronic inflammation when patients complain of recurrent nosebleeds. Although the amount of blood from epistaxis caused by granulation tissue inside the nasal cavity is small, chronic inflammation can cause the development of a pseudoaneurysm and subsequent, massive, life-threatening bleeding. The removal of inflammatory tissue should be performed along with the endovascular treatment of the pseudoaneurysm. Therefore, people who consistently have epistaxis after TSA, even if the bleeding is not in large amount, should be actively screened and treated for nasal chronic inflammation.

## Data Availability

N.a.

## Abbreviations

TSA: Transsphenoidal approach
ICA: Internal carotid artery
MRI: Magnetic resonance imaging
CT: Computed tomography
CSF: Cerebrospinal fluid
MSSA: Methicillin-susceptible staphylococcus aureus
